# Estimates of Cancer Mortality in Hanoi and Ho Chi Minh City, Viet Nam in the 1990s

**DOI:** 10.2188/jea.12.179

**Published:** 2007-11-30

**Authors:** Le Tran Ngoan, Tetsuya Mizoue, Takesumi Yoshimura

**Affiliations:** Department of Clinical Epidemiology, Institute of Industrial Ecological Sciences, University of Occupational and Environmental Health, Japan.

**Keywords:** Viet Nam, cancer mortality, survival, population-based-cancer-registry

## Abstract

As cancer mortality data is not available, a study regarding the real problem of cancer mortality is timely and urgent in Viet Nam. Therefore the aim of the present study was to calculate cancer mortality in the city of Hanoi and Ho Chi Minh. The correlation between cancer mortality to incidence ratios and relative survival probabilities for 23 cancer sites was estimated according to SEER (1973-97), then cancer mortality was calculated from the cancer incidence and cancer survival for 25 cancer sites in each city. Cancer mortality rate for all cancer sites except skin (ASR per 100,000) was 103.9 for males and 52.4 for females in Hanoi, and 93.7 for males and 60.7 for females in Ho Chi Minh. For males, the five most common cancer deaths were cancers of the lung, liver, stomach, colon/rectum, and nasopharynx in both Hanoi and Ho Chi Minh. For females, cancer death in the cervix was uncommon in Hanoi but the most common site in Ho Chi Minh (ASR 2.2 VS. 14.2 per 100,000). The present findings are the first results of cancer mortality from Viet Nam and should be useful for further cancer control programs there.

## BACKGROUND

Mortality statistics are commonly obtained from death certificate, however, death certificate are not available in Viet Nam at present^1^^,^^2^^)^. From 1995-98, the average annual number of deaths that occurred in hospitals from cancer was only 489 throughout the country (hospital based cancer registry). From these registered numbers, the proportion of cancer deaths was 0.1% of all causes of deaths and 1.5% among new cases of cancer^3^^-^^6^^)^. This proportion of deaths from cancer in Viet Nam was much lower than that of the estimated data for developing countries (0.1% VS. 9.0%) in the 1990s^7^^)^. The reason for this uncommon observation data for cancer mortality is that, only a small number of deaths occurred in hospitals nationwide (Annual cancer death number 489) and this caused serious biased data of cancer mortality in Viet Nam^8^^,^^9^^)^. This underestimation of cancer mortality dose not reflect the real public health problems of cancer morbidity and mortality nationwide. Therefore, a study regarding the real problem of cancer mortality is timely and urgent in Viet Nam. The aim of the present study was to calculate cancer mortality in the two cities of Hanoi and Ho Chi Minh in the 1990s.

## MATERIALS AND METHODS

The geographical unit in the present study was two cities: Hanoi and Ho Chi Minh. Cancer mortality was calculated based on 7 age groups from 0-14 to 65+ for given cancer sites. The number of cancer sites examined in the present study was 25: oral cavity (ICD-9 140-5), nasopharynx (147), other pharynx (146, 8, 9), oesophagus (150), stomach (151), colon/rectum (153-4), liver (155), pancreas (157), larynx (161), bronchus and lung (162), connective tissue (171), melanoma of skin (172), breast (174), cervix uteri (180), corpus uteri (182), ovary (183), prostate (185), testis (186), penis (187), bladder (188), brain/nervous system (191-2), thyroid (193), Hodgkin’s disease (201), Non-Hodgkin’s lymphomas (200, 2), and leukemia (204-8). Cancer sites (other than 25 listed above except for skin) were grouped under “others except for skin.” The number of cancer deaths for a specific site was calculated first, then summed up under “all cancer sites except for skin”.

### Cancer Incidence Data from Viet Nam:

Population based cancer registries have been in existence since 1987 in Hanoi City and 1990 in Ho Chi Minh City. The general population covered by the two cancer registries was numbered at 2,115,673 in Hanoi and 4,820,131 in Ho Chi Minh. The initial cancer incidence from Viet Nam were reported from 1991-93 in Hanoi City and from 1995-96 in Ho Chi Minh City^2^^,^^10^^)^. These data were the source for cancer incidences in the present study. To calculate age standardized incidence and mortality rate (ASR) per 100,000 person-year, we use the world population in the present study.

### Cancer Survival Data from Viet Nam:

Data for hospital based cancer survival for 12 cancer sites is available in hospitals in Hanoi and Ho Chi Minh. We decided to use this data from Viet Nam for the present study because the population based cancer survival and relative cancer survival is not available there at present (Table 1). In the city of Ho Chi Minh, the cancer survival data was available for cervical cancer only, therefore, the survival rate for the other 11 cancer sites was referenced from the results in Hanoi and applied to the survival rate in Ho Chi Minh. The 5-year survival rate for the remaining 13 cancer sites and other cancer sites except for skin was referenced from previous results for developing countries and applied to the 5-year survival rate of the 13 cancer sites in both Hanoi and Ho Chi Minh^11^^)^.

**Table 1.** Hospital based cancer survival data in Viet Nam. (#)

Site (ICD-9)	Cancer sites	Number of study subjects completed the follow-up period	1-year survival	3-year survival	5-year survival

			%	%	%
147	Nasopharynx	359			24.2
150	Oesophagus	64		20.3	-
151	Stomach	340			18.0
153-4	Colon/rectum	239			24.7
171	Connective tissue	72			36.1
174	Breast	108			57.4
180	Cervix uteri	3,901 (1)	62.2 (HCM)		-
		452		69.9 (HN)	
183	Ovary	31 (2)		20.0	-
186	Testis	27 (3)			51.9
187	Penis	224 (4)			71.4
188	Bladder	374			64.1
200, 2	NHL	112			33.0

Source: (#)^16^^,^^24^^-^^35^^)^

HCM: Ho Chi Minh City; HN: Hanoi City

The number of follow-up subjects lost for (1): 642, (2): 39, (3): 31, and (4): 113 that was already excluded from the present analysis. For the other remaining cancer sites, the number of follow-up subjects lost was not stated.

Regarding the relative cancer survival between males and females, the estimated 5-year relative cancer survival for developing countries was seen to be slightly higher in males for cancers of the mouth and pharynx, stomach, larynx, and leukemia in comparison with those in females. That was about equal for cancers of the colon/rectum, melanoma of skin, bladder, kidney, and lymphoma but slightly lower for cancers of the esophagus, liver, pancreas, and lung when compared to those in females^12^^)^. Therefore, we decided to use the 5-year relative survival for both sexes to estimate cancer mortality in both males and females in the present study. For the age specific group, 5-year relative survival was found to be almost the same, 70.4% to 69.4%, for the age groups 45 or less, 45-54, 55-64 and 65-74 for all cancer sites excluding lung and bronchus. A fairly lower 5-year relative survival (59.4%) was seen for the age group 75+ when compared to that in the above younger age groups^13^^)^. However, cancer patients aged 75 or over were reported to be the lowest proportion (4.6%) of all cancer patients in Viet Nam^14^^)^. For all cancer sites, the 5-year relative survival rates were observed to be slightly decreased with increased ages (63.3% and 60.3% for the age group 45-54 and 65-74, respectively). Therefore, we decided to use the survival rate for all ages to estimate cancer mortality for 7 age groups from 1-14 to 65+ in the present study.

### Statistical Methods:

The strength of the relationship between cancer mortality to incidence ratios and relative survival probabilities after each given time period, 5, 3, and 1 year since the date of diagnosis was measured. Pearson Correlation Coefficients were calculated by using SAS software on a personal computer^15^^)^. The relationship between variables (M / I ratios and relative survivals: S_i_) was obtained as (M / I ratios) = a + b * (S_i_), then we have M = I * [(a + b * (S_i_)], where “a” and “b” was the Parameter Estimate of the intercept and the survival, respectively.

### The Correlation between the Cancer Incidence and Mortality:

**Figure 1.**
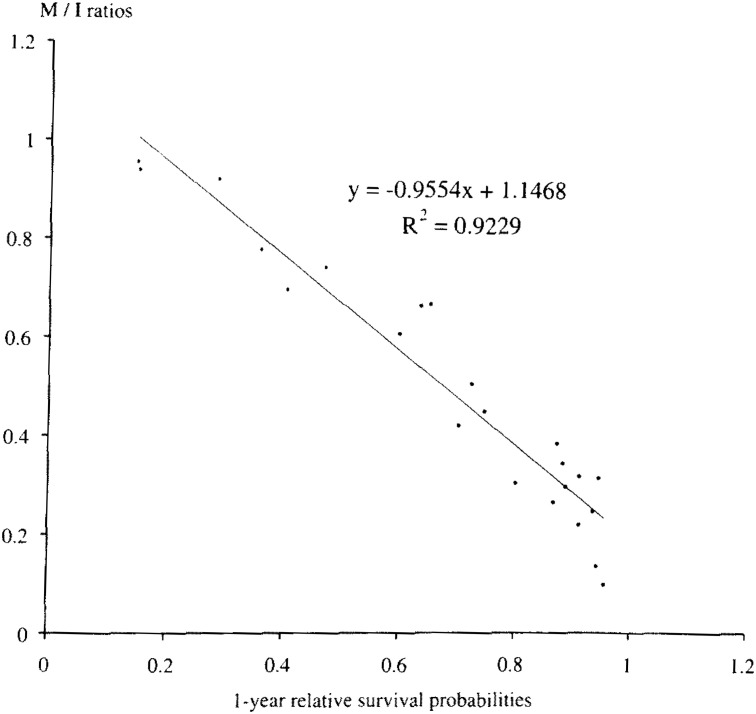
Scatter diagram of data and a trend line for the relationship between mortality/incidence ratios and 1-year relative survival probabilities of SEER for 23 cancer sites.

The present method of estimates of cancer mortality from incidence has been introduced in previous studies^11^^,^^12^^)^. We have applied this method by using the latest data of SEER from 1973-97^13^^)^. Since the survival rate was available in Hanoi and Ho Chi Minh at 5, 3, and 1 year, the scatter diagram of data and a trend line for the relationship between mortality per incidence ratios and 5, 3, 1 year relative survival probabilities was calculated, where y = M : I ratio, x = S_(5)_, S_(3)_, and S_(1)_, respectively. The equations are Y = -0.8717 * X + 0.9219 (R^2^ = 0.9533), Y = -0.8709 * X + 0.9648 (R^2^ = 0.9574), and Y = -0.9554 * X + 1.1468 (R^2^ = 0.9229) for 5, 3, and 1 year relative survival probabilities, respectively. Figure 1 shows the scatter diagram of data and a trend line for the relationship between mortality per incidence ratios and 1-year relative survival probabilities.

## RESULTS

In the male population of Hanoi City during the 3-year period from 1991-93, the number of cancer deaths was 2,506, and the annual mortality rates were 80.3 and 103.9 per 100,000 (crude and ASR rate, respectively). More than one-fourth of cancer deaths was due to lung cancer (26.7%). The second most common cancer death was liver cancer (16.9%), followed by stomach cancer (14.7%) and nasopharygeal cancer (7.1%) (Table 2). For females, the number of cancer deaths was 1,513, and the annual mortality rates were 46.9 and 52.4 per 100,000 (crude and ASR, respectively), the most common cancer death was stomach cancer (15.1%), followed by breast cancer (14.1%), lung cancer (9.8%), and liver cancer (7.9%) (Table 2).

**Table 2.** Number of cancer deaths by age groups in males and females in Hanoi from 1991-93.

Site (ICD-9)	Cancer sites	Number of cancer deaths by age group (year)									Death rate per 100,000	
	
		0-	15-	25-	35-	45-	55-	65	Total	%	Crude	ASR*
Males												
140-5	Oral cavity	1	1	2	6	7	8	9	34	1.36	1.1	1.4
147	Nasopharynx	3	10	8	38	48	45	27	179	7.14	5.7	7.1
146, 8, 9	Other pharynx	0	1	1	5	11	9	14	41	1.64	1.3	1.8
150	Oesophagus	0	0	0	2	8	17	13	40	1.60	1.3	1.8
151	Stomach	0	5	16	46	75	107	120	369	14.72	11.8	15.8
153-4	Colon/rectum	1	3	8	23	39	33	52	159	6.34	5.1	6.8
155	Liver	9	10	22	70	86	129	97	423	16.88	13.5	17.3
157	Pancreas	0	1	3	4	7	15	12	42	1.68	1.3	1.7
161	Larynx	0	0	1	1	3	3	3	11	0.44	0.4	0.5
162	Bronchus and Lung	3	4	10	47	108	263	233	668	26.66	21.4	29.3
171	Connective tissue	5	3	1	5	3	7	9	33	1.32	1.1	1.3
172	Melanoma of skin	1	0	0	2	0	1	0	4	0.16	0.2	0.2
185	Prostate	0	0	0	0	1	6	6	13	0.52	0.4	0.6
186	Testis	1	1	3	1	2	1	0	9	0.36	0.3	0.3
187	Penis	0	1	1	2	2	5	6	17	0.68	0.5	0.7
188	Bladder	0	0	1	1	1	4	7	14	0.56	0.5	0.6
191-2	Brain/nervous system	5	4	2	6	4	3	4	28	1.12	0.9	1.0
193	Thyroid	0	0	2	1	3	2	2	10	0.40	0.3	0.4
201	Hodgkin’s disease	4	2	5	3	2	3	5	24	0.96	0.7	0.8
200, 2	NHL	13	5	15	13	16	21	18	101	4.03	3.3	3.8
204-8	Leukemia	29	16	10	8	9	5	6	83	3.31	2.7	2.7
	Others except for skin	21	18	19	22	37	43	44	204	8.14	6.5	8.0
All cancer sites except for skin		96	85	130	306	472	730	687	2,506	100.00	80.3	103.9
Females												
140-5	Oral cavity	1	1	6	2	6	9	22	47	3.11	1.5	1.6
147	Nasopharynx	1	7	15	14	21	16	13	87	5.75	2.7	3.0
146, 8, 9	Other pharynx	0	0	1	2	2	3	6	14	0.93	0.4	0.5
150	Oesophagus	0	0	1	0	1	3	9	14	0.93	0.4	0.5
151	Stomach	0	5	23	36	34	60	71	229	15.14	7.1	7.9
153-4	Colon/rectum	0	4	8	20	27	24	26	109	7.20	3.4	3.8
155	Liver	3	4	9	15	26	31	31	119	7.87	3.7	4.2
157	Pancreas	0	0	1	2	1	12	3	19	1.26	0.5	0.7
161	Larynx	0	0	0	1	0	0	0	1	0.07	0	0
162	Bronchus and Lung	0	4	10	12	37	36	49	148	9.78	4.6	5.3
171	Connective tissue	4	3	3	4	1	3	5	23	1.52	0.7	0.7
172	Melanoma of skin	0	0	0	0	0	1	1	2	0.13	0.1	0.1
174	Breast	0	2	19	62	73	37	20	213	14.08	6.6	7.6
180	Cervix uteri	0	0	4	16	18	10	12	60	3.97	1.9	2.2
182	Corpus uteri	0	0	1	4	4	3	2	14	0.93	0.4	0.5
183	Ovary	1	3	8	18	9	15	9	63	4.16	2.0	2.1
188	Bladder	0	0	0	0	0	0	1	1	0.07	0.1	0.1
191-2	Brain/nervous system	6	3	3	2	2	2	1	19	1.26	0.6	0.6
193	Thyroid	0	3	4	5	5	6	3	26	1.72	0.8	0.9
201	Hodgkin’s disease	1	2	3	4	3	1	2	16	1.06	0.5	0.5
200, 2	NHL	6	5	6	9	6	11	13	56	3.70	1.7	1.8
204-8	Leukemia	25	13	7	10	12	7	8	82	5.42	2.5	2.7
	Others except for skin	11	9	18	21	23	26	43	151	9.98	4.7	5.1
All cancer sites except for skin		59	68	150	259	311	316	350	1,513	100.00	46.9	52.4

* World population

In the male population of Ho Chi Minh City during the 2-year period from 1995-96, the number of cancer deaths was 2,960, and the annual mortality rates were 64.3 and 93.7 per 100,000 (crude and ASR rate, respectively). The most common cancer death was liver cancer (23.7%), followed by lung cancer (20.9%), stomach cancer (12.8%), and colon/rectum cancer (7.2%). For females, the number of cancer deaths was 2,632, and the annual mortality rates were 52.3 and 60.7 per 100,000 (crude and ASR rate, respectively). Nearly one-fourth of cancer deaths was due to cervical cancer (22.2%), followed by lung cancer (9.3%), stomach cancer (9.2%), colon/rectum cancer (9.1%), breast cancer (8.4%), and liver cancer (8.4%).

**Figure 2.**
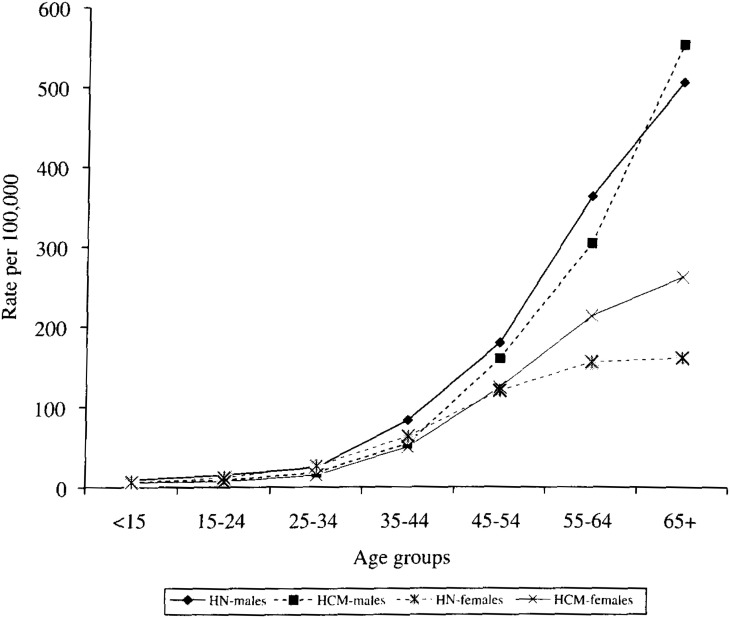
Annual cancer mortality rate by age group in Hanoi and HCM for all cancer sites except for skin in males and females.

*All cancer sites except for skin:* Cancer mortality was higher among males (ASR 103.9 VS. 93.7 per 100,000) but lower among females in Hanoi (ASR 52.4 VS. 60.7 per 100,000) when compared to those in Ho Chi Minh. A fairly higher annual mortality rate was observed for the male age groups 35-44, 45-54, and 55-64 but a much lower annual mortality rate was observed for the female age groups 55-64 and 65+ in Hanoi when compared to those in Ho Chi Minh (Figure 2). However, for both sexes, cancer mortality for all sites except for skin was seen to be similar, that is, about 75 per 100,000 (ASR) and 60 per 100,000 (crude) in both Hanoi and Ho Chi Minh.

*Lung cancer:* For males, lung cancer was the most common cause of death from cancer in Hanoi, the second most common cause in Ho Chi Minh (ASR 29.3 and 20.8 per 100,000, respectively). For females, lung cancer was the third most common cause of death from cancer in Hanoi and the second most common cause in Ho Chi Minh (ASR 5.3 and 5.8 per 100,000, respectively (Table 2, Table 3). Sex ratio (M : F) was 5.5 in Hanoi and 3.6 in Ho Chi Minh. For both sexes, cancer death from lung cancer was the most common cause of death from cancer in Hanoi and the second most common cause in Ho Chi Minh (20.3% and 16.5% of all cancer sites in each city, respectively.

**Table 3.** Number of cancer deaths by age group in males and females in HCM from 1995-96.

Site (ICD-9)	Cancer sites	Number of cancer deaths by age group (year)									Death rate per 100,000	
	
		0-	15-	25-	35-	45-	55-	65-	Total	%	Crude	ASR*
Males												
140-5	Oral cavity	0	0	3	9	12	19	27	70	2.36	1.5	2.3
147	Nasopharynx	0	3	20	21	27	30	20	121	4.09	2.6	3.6
146, 8, 9	Other pharynx	0	1	2	3	7	19	37	69	2.33	1.5	2.3
150	Oesophagus	0	0	0	3	10	26	46	85	2.87	1.9	3
151	Stomach	0	2	14	42	58	93	171	380	12.84	8.3	12.5
153-4	Colon/rectum	1	4	11	32	36	34	95	213	7.20	4.6	6.7
155	Liver	1	16	37	138	134	149	225	700	23.65	15.3	21.8
157	Pancreas	0	0	4	4	9	14	26	57	1.93	1.2	1.9
161	Larynx	0	0	0	2	8	17	37	64	2.16	1.4	2.2
162	Bronchus and Lung	1	3	16	51	73	189	286	619	20.91	13.5	20.8
171	Connective tissue	2	4	5	2	3	1	7	24	0.81	0.5	0.6
172	Melanoma of skin	0	0	0	0	0	0	0	1	0.03	0	0
185	Prostate	0	0	0	0	0	8	29	37	1.25	0.8	1.3
186	Testis	0	0	5	3	1	1	1	11	0.37	0.3	0.3
187	Penis	0	0	1	3	2	2	6	14	0.47	0.3	0.4
188	Bladder	0	1	0	1	1	5	16	24	0.81	0.5	0.8
191-2	Brain/nervous system	11	6	10	9	9	10	6	61	2.06	1.3	1.6
193	Thyroid	0	0	1	2	3	2	5	13	0.44	0.2	0.4
201	Hodgkin’s disease	0	0	2	1	1	1	3	8	0.27	0.1	0.2
200, 2	NHL	10	4	9	6	8	8	16	61	2.06	1.3	1.7
204-8	Leukemia	33	25	18	21	11	2	19	129	4.36	2.8	3.1
	Others except for skin	16	12	15	15	33	41	68	200	6.76	4.4	6.2
All cancer sites except for skin		75	81	173	368	446	671	1146	2,960	100.00	64.3	93.7
Females												
140-5	Oral cavity	0	3	3	2	2	13	39	62	2.36	1.2	1.4
147	Nasopharynx	1	2	9	16	4	10	11	53	2.01	1.1	1.1
146, 8, 9	Other pharynx	1	1	2	1	3	3	8	19	0.72	0.4	0.4
150	Oesophagus	0	0	0	2	0	5	16	23	0.87	0.4	0.5
151	Stomach	0	3	9	21	33	64	113	243	9.23	4.8	5.7
153-4	Colon/rectum	1	1	11	33	33	66	97	242	9.19	4.8	5.6
155	Liver	0	5	11	24	33	51	97	221	8.40	4.4	5.1
157	Pancreas	0	0	0	1	6	12	27	46	1.75	0.9	1.1
161	Larynx	0	0	0	1	0	3	5	9	0.34	0.2	0.2
162	Bronchus and Lung	0	0	3	31	44	61	105	244	9.27	4.8	5.8
171	Connective tissue	2	1	4	3	2	2	2	16	0.61	0.3	0.3
172	Melanoma of skin	0	0	0	0	0	0	1	1	0.04	0	0
174	Breast	0	0	11	59	60	48	43	221	8.40	4.4	5.1
180	Cervix uteri	0	1	22	97	145	201	119	585	22.23	11.6	14.2
182	Corpus uteri	0	0	2	6	10	12	7	37	1.41	0.7	0.9
183	Ovary	3	9	16	25	29	22	29	133	5.05	2.7	3
188	Bladder	0	0	0	1	1	2	6	10	0.38	0.2	0.2
191-2	Brain/nervous system	8	9	7	9	4	4	5	46	1.75	0.9	0.9
193	Thyroid	1	5	6	7	4	5	6	34	1.29	0.7	0.7
201	Hodgkin’s disease	0	0	1	0	1	1	1	4	0.15	0.1	0.1
200, 2	NHL	5	7	4	7	10	10	15	58	2.20	1.2	1.3
204-8	Leukemia	37	11	21	27	11	11	11	129	4.90	2.6	2.7
	Others except for skin	15	10	17	23	26	38	67	196	7.45	3.9	4.4
All cancer sites except for skin		74	68	159	396	461	644	830	2,632	100.00	52.3	60.7

* World population

*Liver cancer:* For males, liver cancer was ranked as the second most frequent cancer in Hanoi but the most frequent cancer in Ho Chi Minh (ASR 17.3 and 21.8 per 100,000, respectively). For females, it was the fourth most frequent cancer in Hanoi and the sixth most frequent cancer in Ho Chi Minh (ASR 4.2 and 5.1 per 100,000, respectively) (Table 2, Table 3). Sex ratio (M : F) was found to be about 4.0 both in Hanoi and Ho Chi Minh. For both sexes, death from liver cancer was the third most common cancer in Hanoi and the most common cancer in Ho Chi Minh (13.5% and 16.5% of all deaths in each city, respectively).

*Stomach cancer:* Stomach cancer mortality rate was the third most frequent cancer in both Hanoi and Ho Chi Minh in males, (ASR 15.8 and 12.5 per 100,000, respectively). For females, stomach cancer death was the most frequent cancer death in Hanoi and the third most frequent cancer in Ho Chi Minh (ASR 7.9 and 5.7 per 100,000, respectively) (Table 2, Table 3). Sex ratio (M : F) was about 2.0 in both Hanoi and Ho Chi Minh. For both sexes, death from stomach cancer was the second most common cause of death from cancer in Hanoi and the third most common cause in Ho Chi Minh (14.9% and 11.1% of all deaths in each city, respectively).

*Nasopharygeal cancer:* For males, nasopharygeal cancer was the fourth most common cause of death from cancer in Hanoi and the fifth most common cause of death from cancer in Ho Chi Minh (ASR 7.1 and 3.6 per 100,000, respectively). For females, it was the sixth most common cause of death from cancer in Hanoi and an uncommon cause of death from cancer in Ho Chi Minh, (ASR 3.0 and 1.0 per 100,000, respectively) (Table 2, Table 3). Sex ratio (M : F) was lower in Hanoi (2.4) than in Ho Chi Minh (3.6).

*Colon/rectum cancer:* In Hanoi, Colon/rectum cancer was the fifth most common cause of death from cancer in both sexes (ASR 6.8 and 3.8 per 100,000, respectively). In Ho Chi Minh, it was the fourth most common cause of death from cancer in both males and females (ASR 6.7 and 5.6 per 100,000, respectively) (Table 2, Table 3).

**Figure 3.**
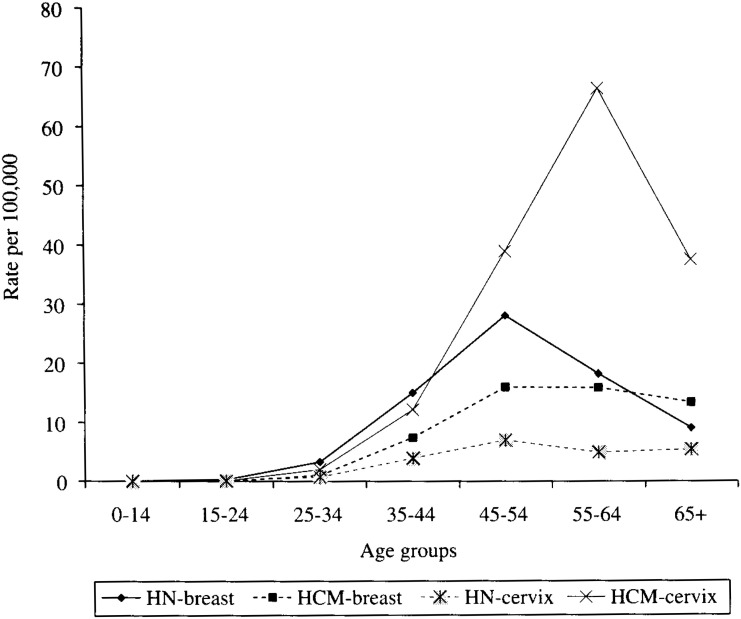
Annual cancer mortality rate by age group in Hanoi and HCM for breast and cervical cancers.

*Breast and cervical cancer:* Breast cancer was the second and fifth most frequent cause of death from cancer in Hanoi and Ho Chi Minh, ASR 7.6 and 5.1 per 100,000, respectively. Cancer death from cervical cancer was an uncommon cause of death from cancer in Hanoi but it was the most frequent cause of death from cancer in females in Ho Chi Minh (ASR 2.2 and 14.2 per 100,000, respectively) (Table 2, Table 3). A much lower annual mortality rate in Hanoi for all specific age groups than in Ho Chi Minh was seen and the highest annual mortality rate in Ho Chi Minh was seen for the age group 55-64 for cervical cancer (Figure 3).

## DISCUSSION

The present findings are the first results of cancer mortality from Viet Nam for the population of Hanoi and Ho Chi Minh in the 1990s. The annual crude mortality from all causes in Hanoi was about 620 per 100,000 in 1991-93 for both sexes^10^^)^. Also for both sexes, the present results have shown that the crude annual mortality rate from cancers at the same time in Hanoi was 63.3 per 100,000. Therefore, the proportion of deaths from cancers was 10.2% of all causes of deaths. The present study findings also present the number of deaths from cancer and its mortality rate for 25 cancer sites in both Hanoi and Ho Chi Minh in males and females. These findings also help us to compare cancer mortality between Hanoi and Ho Chi Minh.

Since the 1950s in both Hanoi and Ho Chi Minh City, cancer treatment and a follow-up for cancer survival in general and cervical cancers in particular has been established^16^^,^^17^^)^. Following the experiences of these researchers (Hien and Hoanh *et al*), cancer treatment and a follow-up has been done well for the 12 cancer sites used in the present study.

Among selected Asian countries where cancer mortality data have been available, for both sexes, the proportion of deaths from cancers in the population of Hanoi from 1991-93 (10.2% of all causes of deaths) was much lower than that in urban China in 1992 (21.8%)^18^^)^. This proportion was also much lower than that in the general population of Japan in 1992 (27.0%), the general population of Hong Kong in 1991 (30.8%), the general population of Singapore in 1991 (24.2%)^19^^-^^21^^)^. This proportion of deaths from cancer was fairly higher than that in the general population of the Philippines in 1993 (7.9%) and in the general population of Thailand in 1994 (9.3%)^22^^,^^23^^)^.

The sex ratios of crude rate and ASR for all cancer sites were somewhat higher in Hanoi than those in Ho Chi Minh (Crude rate: 1.7 VS. 1.2, ASR: 2.0 VS. 1.5) (Table 1, Table 2). These differences may, at least in part, be due to a lower 3-year survival rate for cervical cancer in Ho Chi Minh (16.3%, data not shown) than that in Hanoi (69.9%). Cervical cancer mortality was predominantly high in Ho Chi Minh, comprising about 22.2% of all deaths from cancer, but it was only 4.0% in Hanoi.

However, the present study may have some potential limitations. The relative survival rate was always larger than the observed rate for the same group of patients^13^^)^. Therefore, the number of cancer deaths estimated from observed survival may be, at least in part, overestimated for 12 cancer sites in the present study, which were calculated based on the data of hospital based cancer survival from Viet Nam. These data of observed survival were calculated on the basis of inpatient medical records. The patients with a serious advanced stage of incurable cancer that was diagnosed at outpatient clinics may not be included. Another limitation is data of hospital based cancer survival for 12 cancer sites from Viet Nam and the estimated data for another 13 cancer sites referenced from developing countries. Accuracy of hospital based cancer survival data in Viet Nam is very limited due to the small number of study subjects (Cancers of ovary, testis) and large number of follow-up subjects lost (Cancers of cervical uteri in Ho Chi Minh and penis) (Table 1). In addition estimated data from developing countries that is applied in the present study may not reflect the real problem in Viet Nam. We believe that our population based cancer survival study will provide a better database to calculate cancer mortality in the near future.

In spite of the limitations of the data sources, the present findings have indicated cancer mortality data in the city of Hanoi and Ho Chi Minh that should be useful for further cancer control programs in Viet Nam in particular and in Asia regions in general.

## References

[1] Anh PT, Parkin DM, Hanh NT, Duc NB. Cancer in the population of Hanoi, Vietnam, 1988-1990. Br J Cancer, 1993; 68: 1236-1242. 8260379 10.1038/bjc.1993.511 PMC1968638

[2] Nguyen MQ, Nguyen CH, Parkin DM. Cancer incidence in Ho Chi Minh City, Viet Nam, 1995-1996. Int J Cancer, 1998; 76: 472-479. 9590120 10.1002/(sici)1097-0215(19980518)76:4<472::aid-ijc5>3.0.co;2-o

[3] Ministry of Health Vietnam. Health statistics yearbook. Vietnam Ministry of Health, 1995.

[4] Ministry of Health Vietnam. Health statistics yearbook. Vietnam Ministry of Health, 1996.

[5] Ministry of Health Vietnam. Health statistics yearbook. Vietnam Ministry of Health, 1997.

[6] Ministry of Health Vietnam. Health statistics yearbook. Vietnam Ministry of Health, 1998.

[7] Murray CJ, Lopez AD. Mortality by cause for eight regions of the world: Global Burden of Disease Study. Lancet, 1997; 349: 1269-1276. 9142060 10.1016/S0140-6736(96)07493-4

[8] Matsuda S. An introduction to the health system in Viet Nam. Environ Health Prev Med, 1997; 2: 99-104. 21432462 10.1007/BF02931974 PMC2723538

[9] Lam LH. Health issues and challenge in Vietnam. J Natl Inst Public Health, 1999; 48: 121-133.

[10] Anh PTH, Duc NB, Khang HX, Truong TH, Nga NH. Viet Nam, Hanoi 1991-1993. In; Parkin DM, Whelan SL, Ferlay J, Raymond L, Young J, eds. Cancer incidence in five continents Vol VII, IARC Scientific Publications No. 143, Lyon, IARC, WHO, IACR, 1997: 442-445.

[11] Pisani P, Parkin DM, Bray F, Ferlay J. Estimates of the worldwide mortality from 25 cancers in 1990. Int J Cancer, 1999; 83: 18-29. 10449602 10.1002/(sici)1097-0215(19990924)83:1<18::aid-ijc5>3.0.co;2-m

[12] Pisani P, Parkin DM, Ferlay J. Estimates of the worldwide mortality from eighteen major cancers in 1985. Implications for prevention and projections of future burden. Int J Cancer, 1993; 55: 891-903. 8253525 10.1002/ijc.2910550604

[13] Ries LAG, Eisner MP, Kosary CL et al. SEER cancer statistics review, 1973-1997. National Cancer Institute, Bethesda, Maryland, 2000.

[14] Truong LT. Viet Nam, Ho Chi Minh City, 1976-81. In; Parkin DM, ed. Cancer occurrence in developing countries. IARC Scientific Publications No. 75, Lyon, France, 1986: 309-311.

[15] The SAS System for Windows [program]. Release 6.12 version. SAS Institute Inc, Noth Carolina, USA, 1996.

[16] Hien DB. Analyse de 615 cas de cancer du col uterin vus & traites a L’Institue Radium Hanoi (1955-1961). Med Sci Vietnam, 1962; 4: 72-85. (in Vietnamese with France abstract)

[17] Hoanh DD, Tam PB, Vien NL, Can NH. Cancer of the cervix in South Vietnam. Gann Monogr Cancer Res, 1976; 18: 167-175.

[18] WHO. China: selected rural and urban areas, 1992. World Health Statist Annu, 1994; 1994: B322-B329.

[19] WHO. Hong Kong 1991. World Health Statist Annual, 1993; 1993: D366-D369.

[20] WHO. Japan 1992. World Health Statist Annual, 1993; 1993: D370-D373.

[21] WHO. Singapore 1991. World Health Statist Annual, 1993; 1993: D398-D401.

[22] WHO. Philippines 1993. World Health Statist Annu, 1996; 1996: B680-B683.

[23] WHO. Thailand 1994. WHO statistic information system (http://www.who.int/whosis/) 2001; Mortality Data.

[24] Minh LV, Thinh LP, Dat NV, Phuong LA, Phu TT, Hung NC. Review of 5,034 cases of cervical cancer treated in Cancer Center of HCMC in 5 years 1990-1994. Medical Science of Ho Chi Minh City 1997, Special issue of oncology in 1997. 267-273. (in Vietnamese with English Abstract)

[25] Thoi NH. Facteurs prognostiques principaux du cancer du nasopharynx. J Med Pract Vietnam, 1995; 11-1995: 17-19. (Special issue of oncology in Vietnamese with France abstract)

[26] Huan PD, Van DD. La chirurgie D’exerese dans les cancers de L’oesophage thoracique. J Med Sci Pharm Inf, 1999; 57-59. (Special issue of oncology in Vietnamese with France abstract)

[27] Van DD. Traitment chrurgical des cancers gastrique a L’hospital Viet Duc (1970-1992). Med Sci Vietnam, 1993; 7: 45-50. (Special issue of oncology in Vietnamese with France abstract)

[28] Hung NX, Hung PV, Van DD. Les auteurs raportent une serie retrospective de 206 cancers du rectum, traites a L’hospital Viet Duc, de 1989 a 1996. J Med Sci Pharm Inf, 1999; 79-82. (Special issue of oncology in Vietnamese with France abstract)

[29] Nghi DH, Dong DV. Soft tissue sarcoma in Hospital K, 1977-95. J Med Pract Vietnam, 1995; 11-1995: 54-58. (Special issue of oncology in Vietnamese with English abstract)

[30] Loan DP, Duy KV. Breast cancer among residents of Hanoi City: 5-year survival. Med Sci Vietnam, 1993; 173(7): 107-109. (Special issue of oncology in Vietnamese with English abstract)

[31] Dinh NV, Thuan TV, Phuc ND. Diagnosis and treatment of ovarian cancer in K Hospital 1996-98. J Med Sci Pharm Inf, 1999; 169-171. (Special issue of oncology in Vietnamese with English abstract)

[32] Xuan VV, Hieu NV. Diagnostic et traitement des cancers du testicule, Hospital K Hanoi (1979-88). Med Sci Vietnam, 1993; 173(7): 113-117. (Special issue of oncology in Vietnamese with France abstract)

[33] Nghi DH, Kien V, Ha BM. Survival of 5 years of penile cancers in Hospital K. J Med Sci Pharm Inf, 1999; 172-177. (Special issue of oncology in Vietnamese with English abstract)

[34] Trieu NB, Ky N, Hong NP, Ca VNH, Cu NQ. Contribution au diagnostic precoce des tumeurs dela vessie. J Med Pract Vietnam, 1995; 11-1995: 93-95. (Special issue of oncology in Vietnamese with France abstract)

[35] Duc NB, Mo QT. Non Hodgkin’s lymphomas at Hospital K: clinical presentations and valua of diagnostic methods. J Med Pract Vietnam, 1995; 11-1995: 85-90. (Special issue of oncology in Vietnamese with English abstract)

